# Implementation of Spanish adaptation of the European guidelines on cardiovascular disease prevention in primary care

**DOI:** 10.1186/1471-2296-14-36

**Published:** 2013-03-18

**Authors:** Carlos Brotons, José Maria Lobos, Miguel Ángel Royo-Bordonada, Antonio Maiques, Ana de Santiago, Ángel Castellanos, Santiago Diaz, Juan Carlos Obaya, Juan Pedro-Botet, Irene Moral, Vicenta Lizarbe, Rosa Moreno, Antonio Pérez, Alberto Cordero, Francisco Fornés-Ubeda, Benilde Serrano-Saiz, Miguel Camafort-Babkowski, Roberto Elosua, Susana Sans, Carmen de Pablo, Antonio Gil-Nuñez, Fernando de Álvaro-Moreno, Pedro Armario, Olga Cortés Rico, Fernando Villar, Ángel Lizcano

**Affiliations:** 1 Research Unit, Sardenya Primary Health Care Center-Biomedical Research Institute Sant Pau (IIB-Sant Pau), Barcelona, Spain; 2 Jazmín Primary Health Care Center, Health Regional Service (East Area), Madrid, Spain; 3 National School of Public Health, Institute of Health Carlos III, Madrid, Spain; 4 Manises Health Care Center, Valencia, Spain; 5 Cogolludo Health Care Center, SESCAM, Guadalajara, Spain; 6 Periodistas Primary Health Care Center, Health Regional Service (North Area), Madrid, Spain; 7 Pintores Primary Health Care Center, Health Regional Service (South Area), Madrid, Spain; 8 Chopera Primary Care Center, Health Regional Service (North Area), Madrid, Spain; 9 Department of Medicine, Unidad Docente del IMAS, Hospital del Mar, Barcelona, Spain; 10 Dirección General de Salud Pública, Calidad e Innovación, Ministerio de Sanidad, Servicios Sociales e Igualdad, Madrid, Spain; 11 Angiología y Cirugía Vascular, Hospital Universitario de la Princesa, Madrid, Spain; 12 Servicio de Endocrinología y Nutrición, Hospital de la Santa Creu i Sant Pau, Barcelona, Spain; 13 Department of Cardiology, Hospital Universitario de San Juan, San Juan de Alicante, Alicante, Spain; 14 Salud Laboral de la Policía Local de Valencia, Valencia, Spain; 15 Sociedad Española de Medicina y Seguridad del Trabajo, Sociedad Castellana de Medicina y Seguridad del Trabajo, Madrid, Spain; 16 Servicio de Medicina Interna, ICMID. Hospital Clinic-IDIBAPS, Barcelona, Spain; 17 Institut Hospital del Mar d’Investigacions Mèdiques (IMIM), Barcelona, Spain; 18 Institut d’Estudis de la Salut, Barcelona, Spain; 19 Unidad de Rehabilitación Cardiaca, Servicio Cardiología, Hospital Ramón y Cajal, Madrid, Spain; 20 Unidad de Ictus, Servicio de Neurología, Hospital General Universitario Gregorio Marañón, Madrid, Spain; 21 Servicio de Nefrología, Hospital Universitario Infanta Sofía, San Sebastián de los Reyes, Madrid, Spain; 22 Unit of Hypertension and Vascular risk, Department of Internal Medicine, Hospital General de L’Hospitalet, University of Barcelona, Barcelona, Spain; 23 Health Care Center Canillejas, Madrid, Spain; 24 Escuela Nacional de Sanidad, Instituto de Salud Carlos III, Departamento de Medicina Preventiva y Salud Pública, Facultad de Medicina Universidad Autónoma de Madrid, CIBER de Epidemiología y Salud Pública (CIBERESP), Madrid, Spain; 25 Universidad Rey Juan Carlos, Madrid, Spain

**Keywords:** Cardiovascular disease, Practice guidelines, Risk assessment, Primary care providers, Implementation

## Abstract

**Background:**

The successful implementation of cardiovascular disease (CVD) prevention guidelines relies heavily on primary care physicians (PCPs) providing risk factor evaluation, intervention and patient education. The aim of this study was to ascertain the degree of awareness and implementation of the Spanish adaptation of the European guidelines on CVD prevention in clinical practice (CEIPC guidelines) among PCPs.

**Methods:**

A cross-sectional survey of PCPs was conducted in Spain between January and June 2011. A random sample of 1,390 PCPs was obtained and stratified by region. Data were collected by means of a self-administered questionnaire.

**Results:**

More than half (58%) the physicians were aware of and knew the recommendations, and 62% of those claimed to use them in clinical practice, with general physicians (without any specialist accreditation) being less likely to so than family doctors. Most PCPs (60%) did not assess cardiovascular risk, with the limited time available in the surgery being cited as the greatest barrier by 81%. The main reason to be sceptical about recommendations, reported by 71% of physicians, was that there are too many guidelines. Almost half the doctors cited the lack of training and skills as the greatest barrier to the implementation of lifestyle and behavioural change recommendations.

**Conclusions:**

Most PCPs were aware of the Spanish adaptation of the European guidelines on CVD prevention (CEIPC guidelines) and knew their content. However, only one third of PCPs used the guidelines in clinical practice and less than half CVD risk assessment tools.

## Background

Reducing levels of modifiable cardiovascular risk factors is a key goal in cardiovascular disease (CVD) prevention, and management guidelines are the prime means by which scientific societies aim to achieve this goal. CVD prevention guidelines have been published by a consortium of European societies which aim to promote more intensive cardiovascular risk reduction strategies by encouraging the assessment of an individual’s total CVD risk rather than focusing on individual risk factors [1]. In addition, European guidelines clearly stated the importance of developing national guidance on CVD prevention to suit political, economic, social, and medical circumstances at country level. The Spanish Committee for CVD Prevention (*Comité Español Interdisciplinario para la Prevención Cardiovascular*-CEIPC) was created in June 2000, during the European Forum on Prevention of Coronary Heart Disease in Clinical Practice Regional Follow-up Meeting (Southern Europe). The CEIPC is an official alliance of 15 professional scientific societies supported by the Spanish Ministry of Health. Two national adaptations of the European guidelines for CVD prevention have been developed by CEIPC, the first in 2004 [2] and the second in 2008 [3]. These adaptations have introduced some changes with regard to treatment targets and implementation in order to reflect different social and medical circumstances. Both documents have been published in 8 national journals and disseminated through webpages, scientific meetings and training seminars. There is evidence that guidelines adherence remains low and risk assessment tools are not always used as intended [4,5].

The adoption of guidelines in clinical practice has been related to physician awareness/agreement, self-efficacy, outcome expectancy and practice habits/routines, in addition to patient- and system-related factors [6]. Furthermore, the successful implementation of CVD prevention guidelines relies heavily on primary care physicians (PCPs) providing risk factor evaluation, intervention and patient education. In Spain, it is mandatory for PCPs to complete specialty training in family medicine; however, there are still general doctors working as PCPs without any specialist accreditation.

Although several studies analysed implementation issues in Europe, most were small and non-representative of the general population of doctors working in primary care in each country participating in the survey [7,8]. Moreover, the implementation of local adaptations of European guidelines for CVD prevention has not been formally assessed to date. The aim of this study was to assess the use of the Spanish adaptation of the European guidelines on CVD prevention in clinical practice (CEIPC guidelines) among PCPs, and ascertain whether awareness of and barriers to adoption of the CEIPC guidelines varied according to physician characteristics (age, sex, medical specialty) and work setting.

## Methods

### Design and subjects

A cross-sectional survey on awareness of and barriers to CVD prevention guidelines was conducted among PCPs in Spain between January and June 2011. A random sample, stratified by all the seventeen regions of Spain plus the cities of Ceuta and Melilla, was obtained from the list of primary care physicians working in Spain available at the website of the Spanish Ministry of Health [9]. Assuming an estimated true proportion of 0.5, a maximum acceptable difference of 10% and an α error of 0.05, the required sample size calculated was 1,457 PCPs. The number of physicians selected for each stratum was proportional to their distribution in the database. Consecutive random sampling (with replacement of those who declined to participate when contacted by phone) continued until the target number of physicians in each region agreeing to be interviewed was reached. The questionnaire was sent to those who agree to participate.

Written informed consent was obtained from each participant before the questionnaire was answered. Confidentiality was maintained by data coding to eliminate possible identification of personal information.

### Data collection

Focus groups were used to generate and refine items of the questionnaire used in the survey. The questionnaire was tested for comprehension and usefulness in a pilot study on a small sample of GPs. Data were collected by means of a self-administered questionnaire delivered by mail, with questions regarding awareness of the CEIPC guidelines, risk assessment and barriers to the implementation of recommendations. In addition, information on physician demographic characteristics, medical specialty and work setting was collected. The questionnaire comprised 24 questions, most of which prompted a choice from a list of options, allowing for open answers when appropriate.

### Statistical analyses

Means and standard deviations and percentages and 95% confidence intervals were used to describe the continuous and categorical variables. Comparisons by age (<50 years versus >49 years), sex, setting (rural, suburban versus urban), medical specialty (family medicine versus general medicine), and working or not at an academic teaching centre were made using the chi-square test. A p value less than 0.05 was considered statistically significant. All statistical analyses were made with STATA statistical software (Version 9.2).

Ethical approval was not required in Spain at the time the study was carried out.

## Results

Sixty-seven physicians were excluded from the analysis owing to some missing data on major variables. Thus, the analysis was based on the remaining 1,390 PCPs (56% men, 44% women). The response rate was 33.5%, without significant differences among regions. Characteristics of the survey respondents are provided in Table 1.

**Table 1:** Characteristics of participating physicians (n = 1,390)

	**N**	**mean (SD)**
Age (years)	1390	50 (7.24)
Time in practice (years)	1316	21.61 (8.17)
		**% (95% CI)**
Sex		
Male	759	55.65 (52.96-58.30)
Female	605	44.35 (41.69-47.03)
Specialty		
Family medicine	1041	76.65 (74.31-78.88)
General medicine	299	22.02(19.83-24.31)
Others	18	1.32 (0.78-2.08)
Setting		
Urban	961	70.82 (68.32-73.23)
Suburban	254	18.72 (16.67-20.90)
Rural	142	10.46 (8.89-12.22)
Academic Teaching Centre	594	45.07 (42.36-47.80)

The results of PCP awareness of the CEIPC guidelines are shown in Table 2. More than half the physicians (58%) were aware of and knew the CEIPC guidelines. Of these, 62% used them in clinical practice (36% of all physicians) and 38% stated they were employing a different set of local or international guidelines. Approximately a quarter of physicians (26%) were aware of the CEIPC guidelines but did not know their recommendations, and 16% had never heard of them. Physician awareness and use of CEIPC guidelines in clinical practice differed according to physician specialty, with family doctors being more likely than general doctors to be aware of and use the recommendations (60.1% versus 53.4%, p < 0.001, and 64.21% versus 54.82%, p < 0.03, respectively; data not shown in the table). No differences were observed for age, sex, setting, or working at an academic teaching centre.

**Table 2:** Physician awareness of the CEIPC guidelines (n = 1,390)

	**n**	**%**
Were aware and had knowledge	801	57.6
Used them in clinical practice (n = 781)	486	62.2
Did not use them in clinical practice (n = 781)	295	37.8
Were aware of but did not know recommendations	354	25.5
Never heard of CEIPC guidelines	226	16.3

**Figure 1:**
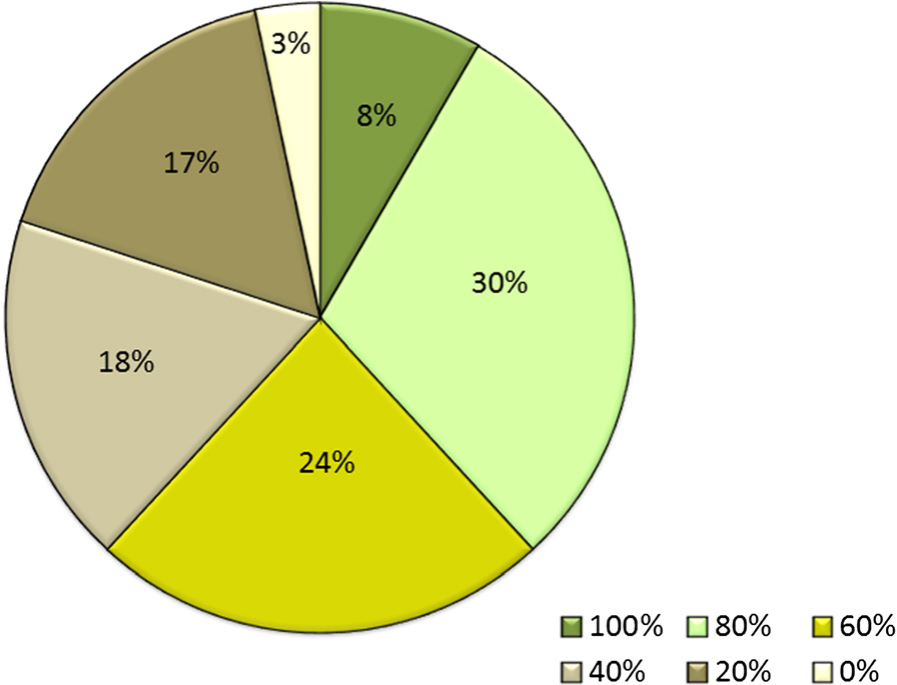
Percentage of patients in whom cardiovascular risk was calculated.

**Figure 2:**
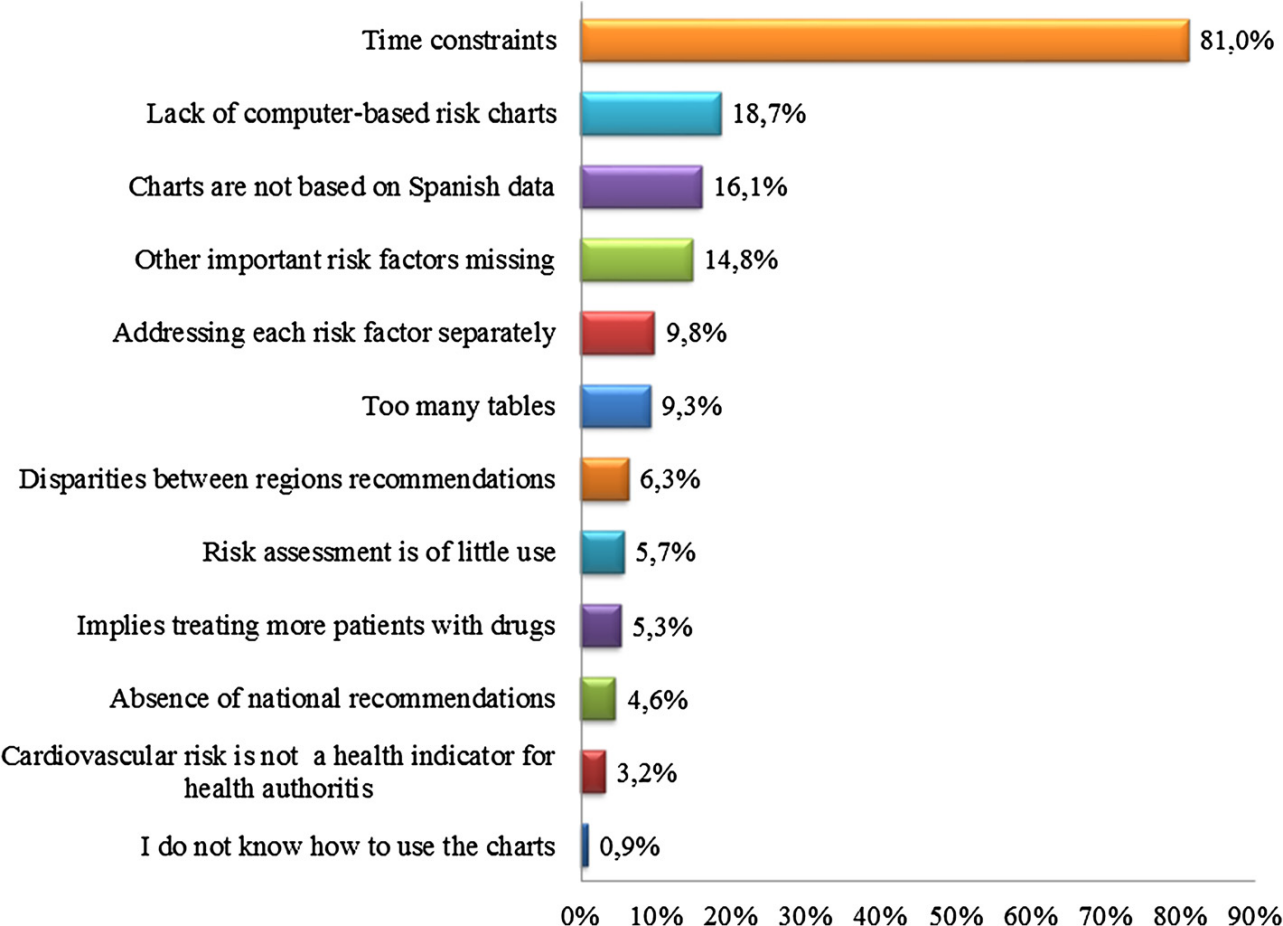
Barriers to the assessment of cardiovascular risk.

Regarding total cardiovascular risk estimation, Figure 1 shows the % of PCPs that calculated the risk in 100%, 80%, 60%, 40%, 20% of the patients or never. We can see that 40% of participants calculated the risk in 80-100% of their patients with at least one risk factor, while 36% of respondents calculated it in 40% or less of their patients. Assessment of CVD risk differed according to physician age (46% of physicians younger than 50 years calculated the risk in 80-100% of patients versus 33% of physicians older than 49 years, p < 0.001), specialty (43% of family doctors calculated the risk in 80-100% of patients versus 23% of general physicians, p < 0.001) and setting (39% of urban physicians calculated the risk in 80-100% of their patients versus 35% of rural physicians, p < 0.001). When PCPs were asked as to the main barrier to assessing cardiovascular risk, the majority of respondents (81%) cited limited surgery time as the most significant (Figure 2). Other barriers mentioned by participants were lack of computer-based risk charts (18%) and charts not being based on Spanish data (16%).

**Figure 3:**
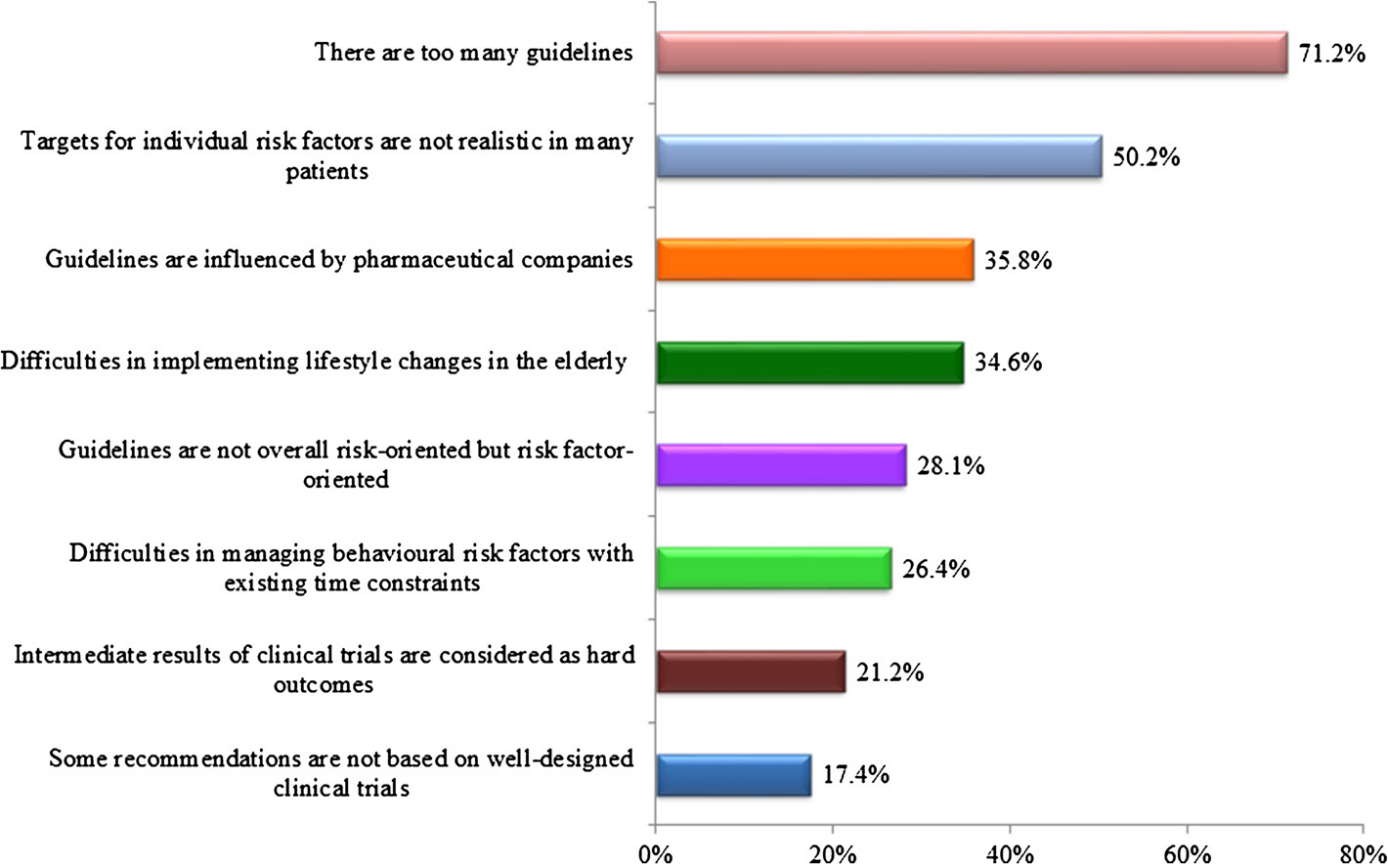
Most common reasons to be sceptical about guidelines recommendations.

When questioned on reasons for being sceptical about the recommendations, 71% of physicians reported that there are too many guidelines, 50% that targets for individual risk factors are unrealistic, and 36% that guidelines are influenced by the pharmaceutical industry (Figure 3).

**Figure 4:**
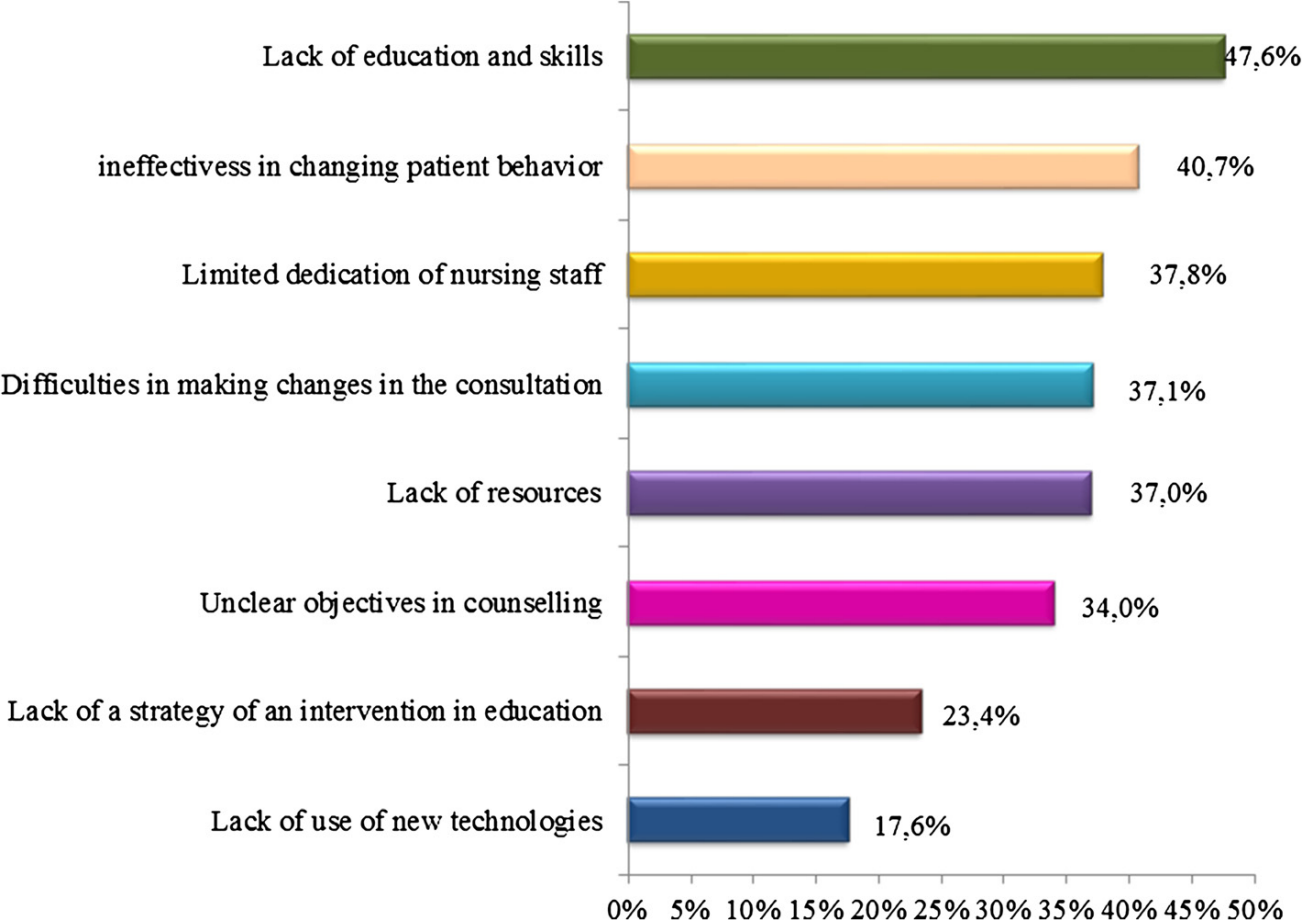
Barriers to the implementation of lifestyle and behavioral change recommendations.

The lack of training and skills was the greatest barrier to the implementation of lifestyle and behavioural change recommendations for 48% of participants. Other barriers commonly cited were the perception that lifestyle advice is not effective in changing patient behaviour (41%), and limited dedication time of nursing staff (38%) (Figure 4).

When PCPs were asked about the reasons why patients sometimes fail to reach the recommended targets for cardiovascular risk factors, 82% stated that patients were the greatest barrier since they do not perceive themselves to be patients at risk, and 63% stated that patients have various comorbidities and treatments (data not shown in the figure).

## Discussion

Our results reflect a wide dissemination of the CEIPC guidelines: more than three quarters of Spanish doctors had heard of the Spanish adaptation of the European guidelines on CVD prevention in clinical practice (CEIPC guidelines) and almost 60% totally or partially knew the contents of the guideline. However, only 36% were using the CEIPC guidelines in clinical practice, with family doctors being more likely to use them than general physicians, as the latter are still working as PCPs without any specialist accreditation. An unexpected result was that PCPs working in academic teaching centres were not doing better in terms of using CEIPC guidelines that those not affiliated to this kind of centres. This result could be explained because CEIPC guidelines were widely disseminated to all kind of centers, regardless their academic characteristics.

A survey of 1,382 physicians from Croatia [10] showed that 56.9% were using the European CVD prevention guidelines in clinical practice; PCPs were found to be more likely to use their own experience, while internists and cardiologists were more likely to use the European guidelines. Another study of 500 physicians from the United States [5] found that, despite 90-100% of awareness of CVD guidelines among physicians, only 50-60% incorporated them into clinical practice. A survey conducted in six European countries among 220 cardiologists and PCPs reported that physicians’ use of CVD prevention guidelines in different European countries varied between 60% and 97% [7].

A discouraging observation stemming from this study is that less than half the physicians (40%) calculated overall cardiovascular risk in more than 80% of their patients with at least one risk factor. Similarly, the *Reassessing European Attitudes about Cardiovascular Treatment* (REACT) study [8], which examined attitudes to and implementation of coronary heart disease and lipid treatment among 754 European PCPs, showed that 43% of physicians rarely or never used risk calculator charts, 43% sometimes referred to them, but only 13% reported that this was always the case. A further survey showed that 62% of physicians used a subjective assessment of risk factors rather than a specific risk calculator, with cardiologists being more likely than PCPs to use a subjective assessment [7]. A recent study done in Canada showed that 74% primary care physicians performed CV risk assessment in eligible patients annually [11]. Interestingly, we found that young, urban and family doctors more often assessed the risk than old, rural and general physicians.

Regarding the barriers to cardiovascular risk assessment, the European survey of physicians’ practices in the control of cardiovascular risks factors (EURIKA study) [12] found that 60% of physicians reported that they did not calculate total cardiovascular risk owing to time constraints, a finding similar to that of our study. A further significant barrier reported in the EURIKA study was that risk assessment is of little use and, moreover the participants did not know how to use it. In contrast, in addition to time constraints, we found the most important barriers to be the lack of computer-based risk charts, charts not being based on Spanish data, major risk factors not being included in risk charts (also reported in the EURIKA study) and each risk factor being addressed separately. Doctors probably do not understand how integrated cardiovascular risk management guidelines still promote the management of raised blood pressure and blood cholesterol. It has been mentioned that it is time for terms such as hypertension and hypercholesterolaemia to be removed from our vocabulary, and the next generation of clinicians should treat risk and not risk factors [13].

Although many physicians in our survey suggested that the patient himself was a significant impediment to achieving CVD targets, this may reflect the perceived difficulty in adherence to lifestyle factors and pharmacological treatments. Other studies [5,8] have also shown patient compliance to be the most common barrier to the implementation of cardiovascular risk reduction. A study designed to assess understanding of CV risk by patients recently diagnosed with diabetes mellitus and/or metabolic syndrome treated at primary care centres showed that participants had poor awareness of their CV risk and almost half thought they had good or excellent health [14].

The most important reason reported by physicians for being sceptical about recommendations was the existence of too many guidelines, a result similar to that found by researchers of the EURIKA study [12]. PCPs may be overwhelmed by the amount of literature they receive and the existence of multiple guidelines for the same topic, which renders it difficult for them to determine which one is/are the best to use in clinical practice. Another important reason reported was that targets for individual risk factors are not realistic in many patients, a fact also mentioned by physicians of the EURIKA study [12]. In clinical practice sometimes it is not easy to implement recommendations of guidelines and achieve specific targets because patients may have multiple comorbidities, being a real challenge to appropriately manage all the conditions.

Almost half the PCPs in our study felt they had insufficient personal training and probably needed further experience and skills to improve the implementation of lifestyle and behavioural change recommendations in their patients. In addition, 41% felt sceptical about the effectiveness of lifestyle advice. This finding is similar to that of a survey of 2,082 PCPs carried out in eleven European countries [15] which reported that 58.2% and 52.8% of physicians had the perception that they were minimally effective in helping patients achieve or maintain normal weight or in helping patients practise regular physical exercise, respectively. Another major barrier to the implementation of lifestyle and behavioural change recommendations reported in our study was the limited dedication time of nursing staff. In another study [7] 46% of physicians recommended increasing the number of nurses trained in prevention as a practical means of improving guideline implementation. It has previously been suggested that clinical practice may be improved by allowing nurses to discuss more thoroughly with patients the importance of lifestyle changes in reducing the risk of CVD [16,17].

One limitation of this study regards low participation rate and sample representativeness. PCPs who responded to the survey presumably were more interested in CVD prevention and aware of the CEIPC guidelines, although the potential biases are unpredictable. There is the potential that nonresponding physicians have a different knowledge base, awareness, practice patterns, and perceptions than our responding physicians. However, the large sample size and the similar regional distribution of nonresponders help mitigate some of this concern. In addition, this study was largely based on self-reporting by PCPs, which might not accurately reflect the way they actually practise.

Another limitation of this study is that we lack information on reliability and validity of the questionnaire used. Whether this has any impact on the results remains to be evaluated. However, we did perform a pilot study in order to test for comprehension and usefulness.

Mean age of respondents (50 years) and mean time in practice (22 years) reflect a population relatively old, which reflects a population of doctors with high experience in clinical practice, and this might have influenced the results of the study. Despite such limitations, we are reasonably confident of the generalizability of our results, as the sample of doctors was large, randomly selected, and represented all the regions of Spain.

## Conclusions

Most Spanish PCPs were aware of the Spanish adaptation of the European guidelines on CVD prevention in clinical practice (CEIPC guidelines) and more than half knew the content of the guidelines. However, the implementation of the guidelines can still improve, given that only one third of PCPs used them in clinical practice and less than half use CVD risk assessment tools. It is important to register relevant information in electronic medical records, rendering automated calculation of risk and the use of on-screen reminders possible. Strategies to overcome organisational constraints such as consultation time, and greater involvement of nurses and other health care professionals should be developed.

## Competing interest

The authors declare that they have no competing interest.

## Authors’ contributions

CB, JML, MAR-B, AM, AC, SD, JCO participated in the conception and design of the study, interpretation of results, drafting the manuscript and revising it critically. CB and IM coordinated the statistical analysis. JP-B, IM, VL, RM, AP, AC, FF-U, B S-S, MC-B, RE, SS, CdP, AG-N, FdA-M, PA, OCR, FV, MAL have been involved in the conception and design of the study, and in revising critically the manuscript for important intellectual content. All authors have given final approval of the version to be published.

## Pre-publication history

The pre-publication history for this paper can be accessed here:

http://www.biomedcentral.com/1471-2296/14/36/prepub
